# Interventions after acute stress prevent its delayed effects on the amygdala

**DOI:** 10.1016/j.ynstr.2019.100168

**Published:** 2019-04-30

**Authors:** Prabahan Chakraborty, Sumantra Chattarji

**Affiliations:** a National Centre for Biological Sciences, Bangalore, 560065, India; b Centre for Brain Development and Repair, Institute for Stem Cell Biology and Regenerative Medicine, Bangalore, 560065, India; c Centre for Discovery Brain Sciences, Deanery of Biomedical Sciences, University of Edinburgh, Hugh Robson Building, 15 George Square, Edinburgh, EH89XD, UK

**Keywords:** Stress, Basolateral amygdala, Anxiety, Dendritic spines, Diazepam, Corticosterone

## Abstract

Stress is known to elicit contrasting patterns of plasticity in the amygdala and hippocampus. While chronic stress leads to neuronal atrophy in the rodent hippocampus, it has the opposite effect in the basolateral amygdala (BLA). Further, even a single episode of acute stress is known to elicit delayed effects in the amygdala. For example, 2 h of immobilisation stress has been shown to cause a delayed increase in dendritic spine density on BLA principal neurons 10 days later in young rats. This is paralleled by higher anxiety-like behaviour at the same delayed time point. This temporal build-up of morphological and behavioural effects 10 days later, in turn, provides a stress-free time window of intervention after exposure to acute stress. Here, we explore this possibility by specifically testing the efficacy of an anxiolytic drug in reversing the delayed effects of acute immobilisation stress. Oral gavage of diazepam 1 h *after* immobilisation stress prevented the increase in anxiety-like behaviour on the elevated plus-maze 10 days later. The same post-stress intervention also prevented delayed spinogenesis in the BLA 10 days after acute stress. Surprisingly, gavage of only the vehicle also had a protective effect on both the behavioural and synaptic effects of stress 10 days later. Vehicle gavage was found to trigger a significant rise in corticosterone levels that was comparable to that elicited by acute stress. This suggests that a surge in corticosterone levels, caused by the vehicle gavage 1 h after acute stress, was capable of reversing the delayed enhancing effects of stress on anxiety-like behaviour and BLA synaptic connectivity. These findings are consistent with clinical reports on the protective effects of glucocorticoids against the development of symptoms of post-traumatic stress disorder. Taken together, these results reveal strategies, targeted 1 h after stress, which can prevent the delayed effects of a brief exposure to a severe physical stressor.

## Highlights

• Acute immobilisation stress increases anxiety and BLA spinogenesis 10 days later.
• Oral gavage of diazepam 1 h *after* stress prevents both these delayed effects.
• Oral gavage of vehicle also has a similar protective effect on anxiety and spines.
• Vehicle-gavage administration leads to an increase in levels of corticosterone.
• This *post-stress* corticosterone surge may have prevented stress-effects 10 days later.

## Graphical abstract

**Figure:**
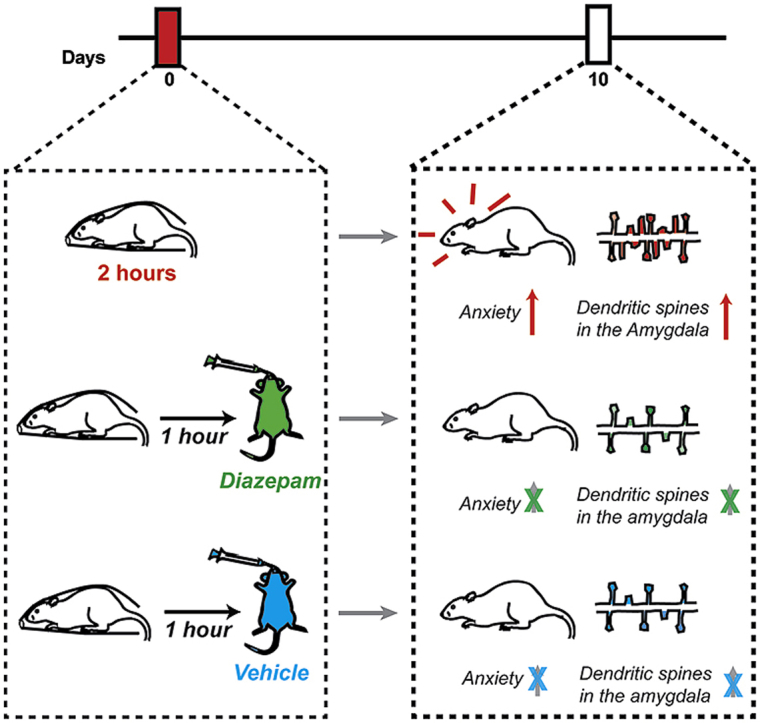

## 1 Introduction

Growing evidence suggests that stress-induced plasticity exhibits contrasting features between the hippocampus and amygdala (Chattarji et al., 2015). For example, exposure to chronic stress causes loss of dendrites and spines in the rodent hippocampus (McEwen, 1999; McEwen et al., 2015; Vyas et al., 2002) along with deficits in spatial memory (Conrad, 2008; Kim and Diamond, 2002; McEwen, 1999). On the other hand, chronic stress enhances dendritic spine density as well as arborisation of principal neurons in the basolateral amygdala (BLA) (Chakraborty and Chattarji, 2018; Mitra et al., 2005; Suvrathan et al., 2014; Vyas et al., 2002). Chronic stress also leads to increased anxiety-like behaviour (Mitra et al., 2005). Interestingly, the duration of stress can modulate the spatiotemporal patterns of spinogenesis in the BLA. For instance, a single 2-h session of immobilisation stress in rodents leads to a significant increase in dendritic spine-density on BLA principal neurons 10 days later (Madan et al., 2018; Mitra et al., 2005; Rao et al., 2012; Yasmin et al., 2016), which is not evident 1 day after stress (Mitra et al., 2005). Moreover, this form of acute stress is also accompanied by an increase in anxiety-like behaviour at the same delayed time point (Chattarji et al., 2015; Mitra et al., 2005; Rao et al., 2012). Together, these findings suggest a unique temporal pattern of spine plasticity in the BLA following exposure to a single episode of immobilisation. Interestingly, this also offers a period of time *after* stress to intervene with an anxiolytic drug to explore the potential prevention of the delayed effects.

Earlier work testing the efficacy of pharmacological interventions focussed primarily on treatment with anxiolytic drugs either during or before the onset of chronic stress (Bondi et al., 2008; Duman et al., 1999; Malberg et al., 2000; Muscat et al., 1992; Nibuya et al., 1996; Stout et al., 2002; Watanabe et al., 1992; Willner et al., 1987). More recent work has used acute stress paradigms to address the same question. Interestingly, such interventions at time points *after* stress have been shown to prevent enhanced anxiety-like behaviour (Cohen et al., 2011, 2006; Matar et al., 2009, 2006; Zohar et al., 2011, 2008) and reduced spine density in the hippocampus (Zohar et al., 2011). However, it is not known if such interventions can also prevent the opposite effects of stress in the BLA, one of the key loci involved in the regulation of anxiety-like behaviour (Adhikari, 2014; Felix-Ortiz and Tye, 2014; Tye et al., 2011). Moreover, previous studies that report prevention of acute stress effects on the BLA have used pharmacological interventions either before (Rao et al., 2012) or during stress exposure (Yasmin et al., 2016). From a translational perspective, pharmacological interventions *after* stress bear more clinical relevance, especially for conditions like post-traumatic stress disorder (PTSD), wherein increase in anxiety is both delayed and prolonged, and is accompanied by amygdalar hyperactivity (Koenigs and Grafmann, 2009; Rauch et al., 2006; Shin and Liberzon, 2010; Stillman and Aupperle, 2014).

Therefore, in the present study young rats were administered systemic doses of the anxiolytic diazepam 1 h *after* a single episode of immobilisation stress to test if such a post-stress intervention is able to prevent its delayed impact on amygdalar spinogenesis and anxiety-like behaviour 10 days later.

## 2 Materials and methods

### 2.1 Animals

Young male Wistar rats from Charles River laboratories, ∼60 days old at the beginning of the experiment, were used for this study. Animals were housed in groups of 2 animals per cage with *ad libitum* access to food and water, and maintained on a 14 h: 10 h light: dark cycle in a temperature controlled environment. All efforts were made to minimise animal suffering and to reduce the number of animals used. All maintenance and experimental procedures were approved by the Institutional Ethics Committee, National Centre for Biological Sciences, India. A total of 159 animals were used in this study (Number of animals used: *For morphological analysis*: Control (no gavage) N = 6, Stress (no gavage) N = 6, Control + Vehicle, N = 4, Stress + Vehicle, N = 4, Control + Diazepam, N = 4, Stress + Diazepam, N = 4; *For behavioural analysis*: Control (no gavage) N = 12, Stress (no gavage) N = 11, Control + Vehicle, N = 20, Stress + Vehicle, N = 25, Control + Diazepam, N = 19, Stress + Diazepam, N = 24; *For ELISA*: Baseline, N = 7, After gavage, N = 8, After stress, N = 5).

### 2.2 Stress procedure

Prior to stress, animals were handled for a period of three days, and then randomly assigned to the acute immobilisation stress (stress) and control groups. Stress was done as previously described (Madan et al., 2018; Mitra et al., 2005; Rao et al., 2012). Briefly, animals were immobilized in plastic immobilisation cones with no access to food or water for 2-h, only on a single day. Anxiety and dendritic spines were quantified 10 days later. Animals were not handled within this duration. All stress protocols were carried out between 10 a.m. and 12 p.m. Control rats were not subjected to any stress, and were housed in a room different from the stressed animals. Different batches of animals were used for morphology, behaviour and corticosterone quantification experiments.

### 2.3 Golgi-Cox staining

10 days after stress, stressed and control animals were decapitated after halothane anaesthesia and brains were removed for modified Golgi-Cox staining, as described previously (Chakraborty and Chattarji, 2018; Madan et al., 2018; Patel et al., 2018). The staining protocol was a modified version of traditional Golgi-Cox staining technique. Briefly, brains were incubated in Golgi-Cox solution composed of mercuric chloride, potassium chromate and potassium dichromate, and the solution was changed after 24 h. Subsequently, samples remained incubated for a total duration of 14 days, followed by 5% sucrose in 0.5 M phosphate buffer for 5 days. Finally, 120 μm thick serial coronal sections were collected on gelatin-chrome alum coated slides using a Leica vibratome (VT-1200S). Sections were developed in 5% sodium carbonate, dehydrated in grades of alcohol and finally mounted with DPX (Nice Chemicals, India).

### 2.4 Dendritic spine analysis

Prepared slides were coded and quantification of dendritic spine on apical dendrites of BLA pyramidal neurons was done blind. For this study, the apical dendrite originating directly from the cell body was considered as a main shaft, and dendrites branching off from the main shaft were considered as primary dendrites. Neurons were chosen for analysis based on criteria described earlier (Mitra et al., 2005; Rao et al., 2012). Pyramidal neurons in the BLA were classified based on their morphological characteristics, which include a triangular shaped soma, a single, distinct apical dendrite, and at least two basal dendritic branches (Hill et al., 2013). Also, our analysis was restricted to medium spiny neurons (Hill et al., 2013; Madan et al., 2018; Mitra et al., 2005; Rao et al., 2012). Each primary dendrite chosen was analysed for a length of 80 μm from its origin, and required to be (i) untruncated, (ii) consistent impregnation along its entire length, and (iii) isolated from and not overlapping with neighbouring neurons. All protrusions from the primary dendrite were considered as spines, irrespective of their morphological characteristics. An average of 6 dendrites were analysed per animal. Analysis was performed using Neurolucida image analysis software from Micro-BrightField, Williston, VT, USA, attached to an Olympus BX61 microscope (100×, 0.95 Numerical Aperture, Olympus BX61) from Olympus, Shinjuku-Ku, Tokyo, Japan.

### 2.5 Elevated plus-maze

Animals were subjected to elevated plus-maze 10 days after acute stress. Light intensities at the end of the open-arm, centre of the maze and throughout the closed arm were approximately 80 lx, 10 lx and 0.2 lx, respectively. Prior to behavioural testing, all animals were transported to a holding room and acclimatised for 20 min. Subsequently, animals were placed in the centre of the maze along the diagonal with a free choice to explore the open or the closed arm. Each animal was allowed to explore the maze for 5 min. All trials were conducted between 10 a.m. and 2 p.m. and videotaped for offline analysis. Number of entries into the open and closed arms and time spent in the open-arm was analysed while being blind to the treatment. Open-arm exploration was measured by normalizing to total exploration on the maze, with a reduction in open-arm exploration indicating increased anxiety. Anxiety Index, a measure that takes into account both open-arm time and entries, was calculated as previously described (Rao et al., 2012), using the following equation:

$$\mathrm{Anxiety}\;\mathrm{Index}\;=1-[\;(\frac{\mathrm{Open}\;\mathrm{Arm}\;\mathrm{Time}}{\mathrm{Total}\;\mathrm{Time}}+\frac{\mathrm{Number}\;\mathrm{of}\;\mathrm{Open}\;\mathrm{Arm}\;\mathrm{Entries}}{\mathrm{Total}\;\mathrm{Entries}})/2]$$

### 2.6 Drug treatment

Diazepam (gift by Hikal India Ltd.) was dispersed in a vehicle of 1% Tween 20 (v/v) in 0.9% saline (w/v), by suspending it in a vortex, to finally constitute a concentration of 2 mg/ml. All animals got a single, oral gavage of 5 mg/kg body weight dose of diazepam or vehicle, 1 h after the end of immobilisation stress. For example, an animal weighing 320 gm received 0.8 ml of either vehicle or diazepam (2 mg/ml), depending on the experimental group it was allotted to.

### 2.7 Corticosterone ELISA

Corticosterone ELISA was performed as previously described (Madan et al., 2018). Briefly, animals were anesthetised by halothane, and were subsequently decapitated under deep anaesthesia. Trunk blood was collected from different cohorts of rats after handling (baseline), after oral gavage of vehicle or after immobilisation stress. Blood samples were centrifuged at 4 °C, and supernatant serum was stored in a separate vial at −20 °C until analysis. Corticosterone ELISA was performed using a kit (Enzo Life Sciences, ADI-900-097) following kit instructions. Two plates were run for the corticosterone ELISA assay. The intra-assay variability was 6.78% and 4.82% for plates 1 and 2 respectively. The inter-assay variability was 5.8%.

### 2.8 Statistical analyses

All values are reported as Mean ± SEM. Each data set was evaluated for outliers, which was defined as greater than twice the standard deviation away from the mean. Student's unpaired, two-tailed *t*-test was used for analyses of all behavioural parameters as well as for comparing total spine densities in the baseline (no gavage) groups. Additionally, a 2-way ANOVA was used for comparing among the gavage-treated groups, followed by *post-hoc* Tukey's test. Further, a 2 × 3 ANOVA was used to compare between the gavage and no gavage groups, followed by *post-hoc* Dunnett's test to compare all other experimental groups against the stress (no gavage) group. For analysis of corticosterone ELISA data across time points, one-way ANOVA was used, followed by Tukey's test for *post-hoc* analysis. All datasets were subjected to homoscedasticity test. All statistical analyses and plots were done using GraphPad Prism software (GraphPad software Inc., La Jolla, California, USA, version 6). The figure panels were made with Adobe Creative Design Suite, version 5.

## 3 Results

### 3.1 Acute stress leads to a delayed increase in anxiety-like behaviour

**Fig. 1:**
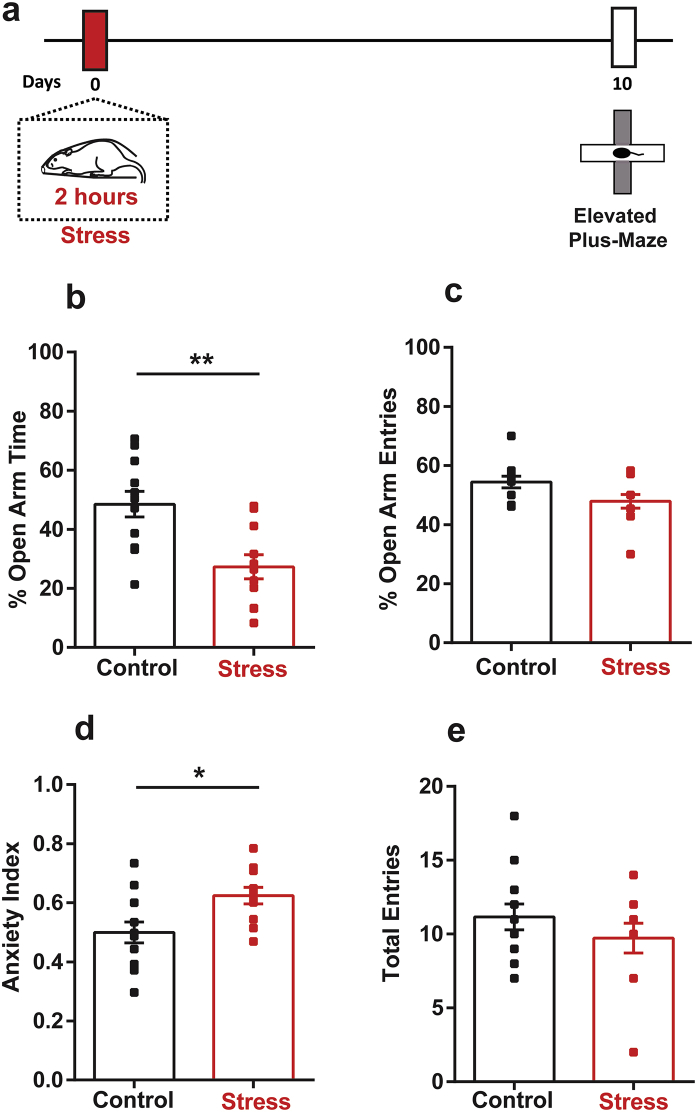
**Stress causes a delayed increase in anxiety-like behaviour. (a)** Experimental design. Rats underwent a single 2-h session of acute immobilisation stress, and anxiety-like behaviour was measured on the elevated plus-maze 10 days later. Control animals remained unstressed. **(b)** Stressed animals spend significantly less time in the open-arm. **(c)** Stressed animals show a trend towards decreased open-arm entries that did not reach statistical significance. **(d)** Stress leads to a delayed increase in the Anxiety Index. **(e)** Stress did not affect total entries on the maze. (Control, N = 12 animals; Stress, N = 11 animals). ** indicates p < 0.01 and * indicates p < 0.05 in Student's unpaired *t*-test.

A single 2-h episode of immobilisation stress is known to cause a delayed increase in anxiety-like behaviour on the elevated plus-maze in young rats (Mitra et al., 2005; Rao et al., 2012). We first wanted to reconfirm the same behavioural effect of acute stress in the present study by testing stressed animals on the plus-maze 10 days later (Fig. 1a). Consistent with earlier reports, stressed animals spent significantly less time in the open-arm compared to unstressed controls (*Control:* 48.51 ± 4.32%, *Stress:* 27.3 ± 4.11%; t_21_ = 3.54, *p* < *0.01*) (Fig. 1b) indicating enhanced anxiety-like behaviour. The decrease in number of open-arm entries, however, did not reach statistical significance (*Control:* 51.54 ± 3.39%, *Stress:* 47.88 ± 2.32%; t_21_ = 0.87, p = 0.39) (Fig. 1c).

Next, we calculated an Anxiety Index that takes into account both the time spent in the open-arm as well as the number of entries made into it (*Materials and Methods,* Rao et al., 2012). An index value closer to 1 indicates higher anxiety, while a value closer to 0 indicates lower anxiety. Stressed animals showed a higher value of Anxiety Index relative to unstressed controls (*Control:* 0.5 ± 0.04, *Stress:* 0.62 ± 0.03; t_21_ = 2.73, *p* < *0.05*) (Fig. 1d). Lastly, the total number of entries into the open and closed arms, which is used as a measure of locomotor activity on the maze (Walf and Frye, 2007), was not affected by stress (*Control:* 11.17 ± 0.88, *Stress:* 9.73 ± 1.00; t_21_ = 0.87, p = 0.29) (Fig. 1e).

### 3.2 Acute stress leads to a delayed increase in dendritic spine density in the basolateral amygdala

**Fig. 2:**
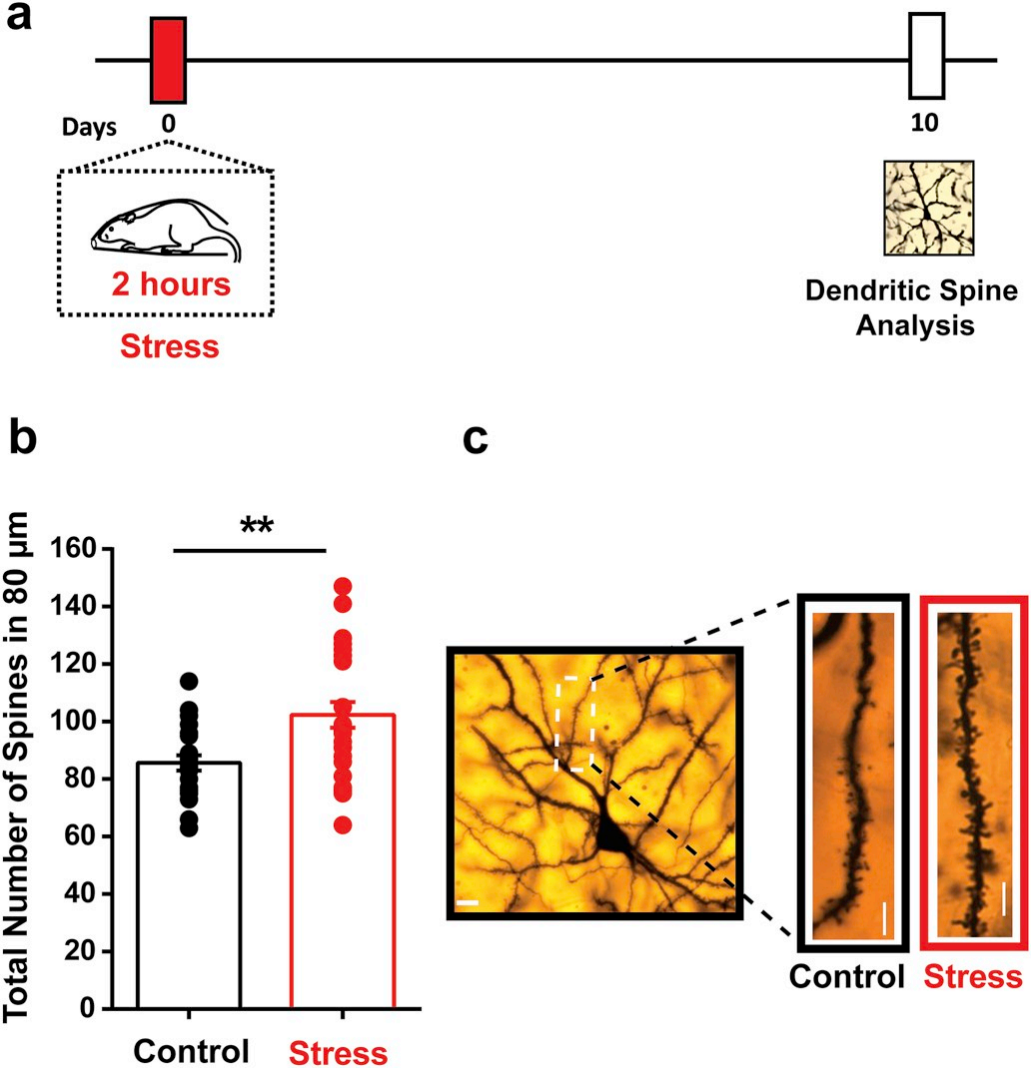
**Stress triggers a delayed increase in dendritic spine density in the basolateral amygdala. (a)** Experimental design. Animals underwent a single 2-h session of acute immobilisation stress, and they were sacrificed 10 days later for Golgi-Cox staining to visualise dendritic spines on the principal neurons of the basolateral amygdala (BLA). Control animals remained unstressed. **(b) S**tress leads to a delayed increase in dendritic spine density in the apical dendrites of the BLA pyramidal neurons. (Control, n = 22 dendrites, N = 6 animals; Stress, n = 28 dendrites, N = 6 animals). (c) Representative images of dendritic segments analysed in the control (*left*) and stressed (*right*) animals. Scale bar 10 μm ** indicates p < 0.01 in Student's unpaired *t*-test.

In addition to the delayed anxiogenic effects of acute stress, it is also known to elicit an increase in density of dendritic spines in the principal neurons of basolateral amygdala (BLA) at the same delayed time point (Mitra et al., 2005; Rao et al., 2012). Therefore, as acute stress led to a delayed increase in anxiety-like behaviour in the present study, a separate cohort of animals was sacrificed 10 days after acute stress to quantify dendritic spine density on BLA principal neurons (Fig. 2a). Acute stress significantly enhanced dendritic spine density on the primary apical dendrites of BLA principal neurons (*Control:* 85.64 ± 2.66, *Stress:* 102.4 ± 4.499; t_48_ = 2.99, *p* < *0.01*) (Fig. 2b). Thus, consistent with earlier reports (Mitra et al., 2005; Rao et al., 2012), a single 2-h exposure to immobilisation stress enhanced both BLA spine density and anxiety-like behaviour 10 days later.

### 3.3 Oral gavage of diazepam as well as vehicle prevents the delayed increase in anxiety-like behaviour

**Fig. 3:**
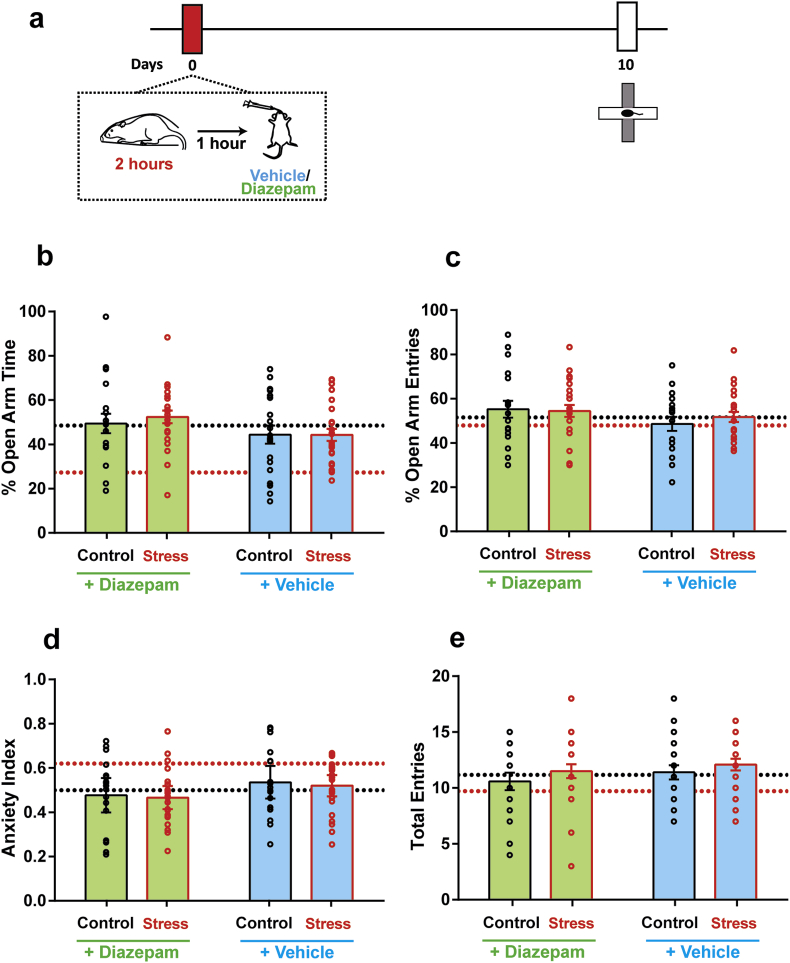
**Diazepam and vehicle oral gavage, 1 h after stress, both prevent anxiety-like behaviour 10 days later. (a)** Experimental design. Animals underwent a single 2 h episode of immobilisation stress (Stress) or remained unstressed (Control), and received either a single dose of diazepam or vehicle gavage 1 h after stress. Anxiety on the elevated plus-maze was quantified 10 days later. **(b)** Open-arm time, **(c)** Open-arm entries and **(d)** Anxiety Index are comparable across all four groups. **(e)** All groups show comparable locomotion on the maze as well (*Control + Vehicle*: N = 20 animals, *Control + Diazepam*: N = 25 animals, *Acute Stress + Vehicle*: N = 18 animals, *Acute Stress + Diazepam*: N = 22 animals). Dotted lines indicate mean values of each parameter in control (no gavage) (*black*) and stressed (no gavage) (*red*) groups. (For interpretation of the references to colour in this figure legend, the reader is referred to the Web version of this article.)

Interestingly, the delayed manifestation of these stress-induced neuronal and behavioural changes offers a stress-free time window of intervention *after* stress. Specifically, this allows us to test the efficacy of a post-stress treatment with an anxiolytic drug in reversing the delayed effects of acute stress. To this end, young rats received a single systemic dose of diazepam via oral gavage, 1 h after acute stress (Fig. 3a), following which they were returned to their home cages. A necessary control for these diazepam experiments is to test the impact of vehicle alone. Thus, a separate group of animals received oral gavage of vehicle 1 h after the same 2-h immobilisation stress. 10 days later, anxiety-like behaviour on the elevated plus-maze was assessed in both control and stressed animals who received either diazepam or vehicle. We expected that the delayed effects of stress would also be seen in the vehicle-treated stressed animals, as the anxiolytic diazepam was completely absent in vehicle.

While stress in itself led to increased anxiety-like behaviour on day 10 (Fig. 1), a single dose of diazepam successfully prevented this increase. Compared to diazepam-treated controls, stressed rats treated with diazepam spent the same amount of time in the open-arm (*Control + Diazepam:* 44.83 ± 3.99%; *Stress + Diazepam:* 52.34 ± 2.05%) (Fig. 3b, *diazepam*). Surprisingly, oral gavage of vehicle *also* had a protective effect similar to diazepam treatment. Stressed and control animals treated with vehicle showed no difference in open-arm exploration – both groups spent similar durations in the open-arm (*Control + Vehicle:* 44.38 ± 4.06%; *Stress + Vehicle:* 44.30 ± 2.72%) (Fig. 3b, *vehicle*). Furthermore, animals across all treatment groups had comparable number of open-arm entries as well (*Control + Diazepam:* 55.69 ± 3.85%; *Stress + Diazepam:* 54.97 ± 2.64%, *Control + Vehicle:* 48.58 ± 3.17%; *Stress + Vehicle:* 51.72 ± 2.24%) (Fig. 3c). Consequently, all treatment groups also showed comparable values of Anxiety Index (*Control + Diazepam:* 0.48 ± 0.04; *Stress + Diazepam:* 0.46 ± 0.02, *Control + Vehicle:* 0.54 ± 0.04; *Stress + Vehicle:* 0.52 ± 0.02) (Fig. 3d). Together, these observations suggested that both vehicle and diazepam gavage prevented the delayed increase in anxiety due to acute immobilisation.

Next, we compared the total number of open and closed arm entries to validate that none of these effects were a result of locomotor differences due to stress or drug treatment. Indeed, all four groups showed comparable numbers of total entries into the open and closed arms of the plus-maze (*Control + Diazepam:* 10.58 ± 0.78; *Stress + Diazepam:* 11.27 ± 0.65, *Control + Vehicle:* 11.40 ± 0.64; *Stress + Vehicle:* 12.08 ± 0.52) (Fig. 3e).

Analysis of each of the above parameters by a two-way ANOVA showed no effects of stress (*Open-Arm Time:* F_(1,82)_ = 1.33, p = 0.25, *Open-Arm Entries:* F_(1,82)_ = 0.17, p = 0.68, *Anxiety Index:* F_(1,82)_ = 0.75, p = 0.39, *Total Entries:* F_(1,82)_ = 1.57, p = 0.21) or diazepam (*Open-Arm Time:* F_(1,82)_ = 1.73, p = 0.19, *Open-Arm Entries:* F_(1,82)_ = 3.095, p = 0.08, *Anxiety Index:* F_(1,82)_ = 2.72, p = 0.10, *Total Entries:* F_(1,82)_ = 1.20, p = 0.28) for any of the parameters. Furthermore, there was also no interaction between the two factors for any of the parameters quantified (*Open-Arm Time:* F_(1,82)_ = 1.39, p = 0.24, *Open-Arm Entries:* F_(1,82)_ = 0.43, p = 0.51, *Anxiety Index:* F_(1,82)_ = 0.11, p = 0.74, *Total Entries:* F_(1,82)_ = 0.04, p = 0.85).

Finally, in order to understand if gavage administration, irrespective of its pharmacological nature, had any effect on anxiety-like behaviour when compared to the groups which did not receive oral gavage (Fig. 1, referred here as the ‘no gavage’ groups), we reanalysed the data across all experimental groups. As time spent exploring the open-arm was most significantly reduced as a result of acute immobilisation (Fig. 1b), we chose this parameter for our present analysis. A 2 × 3 ANOVA revealed a significant main effect of gavage (F_(2,105)_ = 5.24, *p* < *0.01*) without any effects of stress (F_(1,105)_ = 3.76, *p* = 0.06). However, the interaction between both factors was significant (F_(1,105)_ = 4.76, *p* < *0.05*), suggesting that gavage administration indeed altered the effects of stress exposure. This was further confirmed upon *post-hoc* analysis, wherein time spent in the open-arm for the stress (no gavage) group was significantly lower (Fig. 3b, *red dotted line*) than all animals which received gavage as well as in comparison with the control (no gavage) group (Fig. 3b, *black dotted line*) (Table 1, *open-arm time*). In contrast, comparing total entries on the maze showed neither showed any effects of gavage (F_(2,105)_ = 1.49, p = 0.23) or stress (F_(1,105)_ = 0.008, p = 0.93), nor any interaction between both factors (F_(2,105)_ = 1.27, *p* = 0.29). Furthermore, *post-hoc* comparisons revealed no difference between the stress (no gavage) group and all other experimental cohorts (Table 1, *total entries*), implying that the protective effects of oral gavage was not due to locomotor differences on the plus-maze.

**Table 1:** *Post-hoc* comparison of open-arm time, total entries and BLA spine density, of control (no gavage) and all gavage groups, with stress (no gavage) group, following 2 × 3 ANOVA. * indicates p < 0.05, ** indicates p < 0.01, *** indicates p < 0.0001 and **** indicates p < 0.00001.

	compared to Stress (no gavage)				
Parameters	Control *(no gavage)*	Control + Vehicle	Stress + Vehicle	Control + Diazepam	Stress + Diazepam
Open-Arm Time	**	*	*	**	***
Total Entries	p = 0.67	p = 0.44	p = 0.13	p = 0.91	p = 0.36
BLA spine density	**	****	****	****	****

Therefore, these observations suggest that while stress itself led to a delayed increase in anxiety-like behaviour, this was prevented by a single dose of diazepam, as well as a single episode of vehicle gavage administered 1 h after stress.

### 3.4 Oral gavage of diazepam and vehicle, 1 h after stress, *also* prevents spinogenesis in the BLA 10 days later

**Fig. 4:**
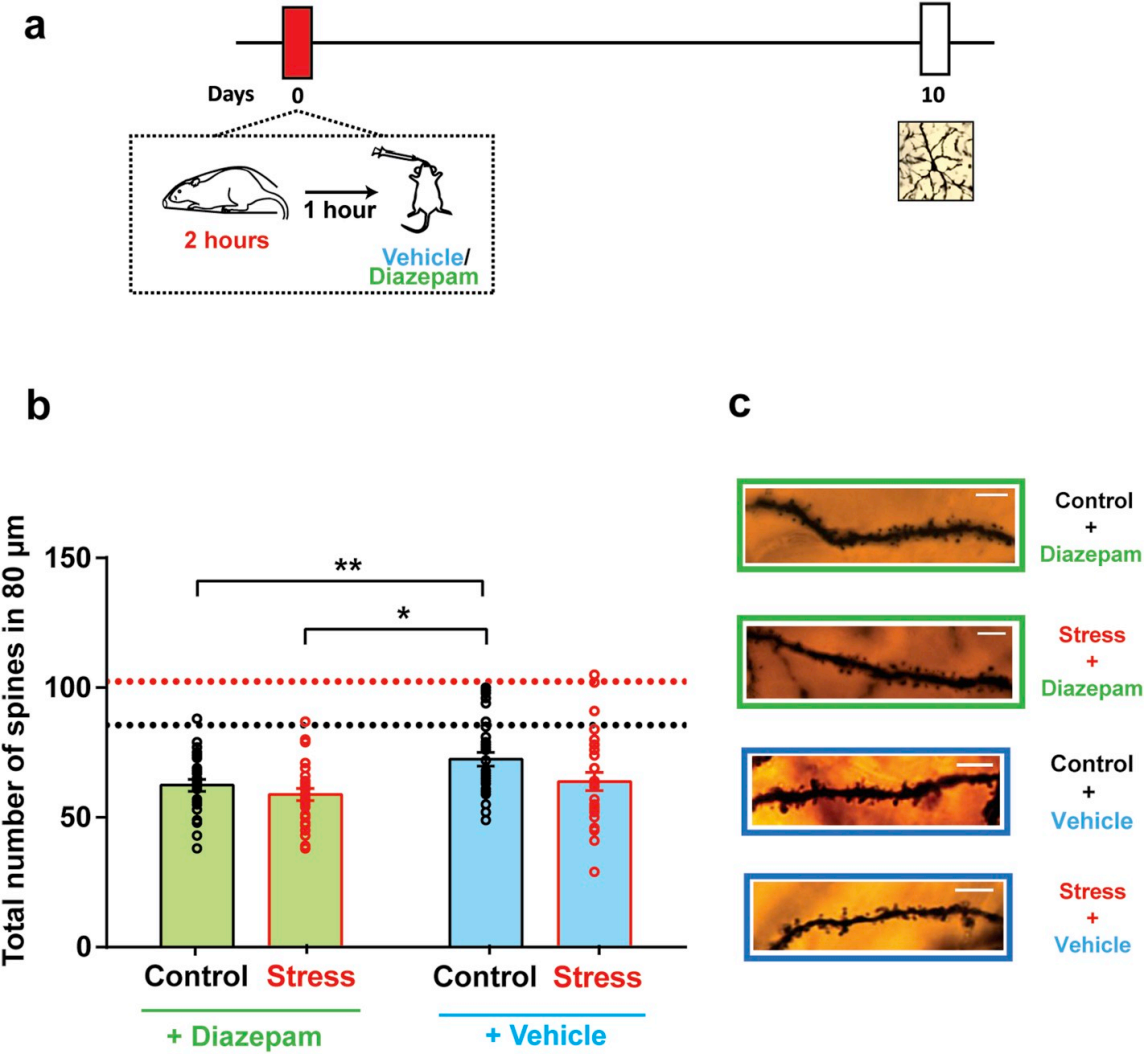
**Diazepam and vehicle oral gavage, 1 h after stress, both prevent the delayed increase in BLA spinogenesis. (a)** Experimental design. Animals underwent a single 2 h episode of immobilisation stress (Stress) or remained unstressed (Control), and received either a single dose of diazepam or vehicle gavage 1 h after stress. Dendritic spine density in the BLA was quantified 10 days later. **(b)** Diazepam and vehicle both prevent spinogenesis in the BLA. (*Control + Vehicle*: n = 32 dendrites, N = 4 animals, *Control + Diazepam*: n = 28 dendrites, N = 4 animals, *Acute Stress + Vehicle*: n = 27 dendrites, N = 4 animals, *Acute Stress + Diazepam*: n = 29 dendrites, N = 4 animals). **(c)** Representative apical dendritic segments from BLA pyramidal neurons from all four groups. Dotted lines indicate mean spine density of control (no gavage) (*black*) and stressed (no gavage) (*red*) groups. ** indicates p < 0.01 and * indicates p < 0.05 in *post-hoc* Tukey's test. (For interpretation of the references to colour in this figure legend, the reader is referred to the Web version of this article.)

In light of the efficacy of post-stress gavage in preventing the delayed anxiogenic effects of stress, we next compared its effect on dendritic spine density in the BLA. Indeed, oral gavage of diazepam, 1 h after acute stress, also successfully prevented the delayed increase in dendritic spine density on BLA principal neurons (*Control + Diazepam:* 62.39 ± 2.31; *Stress + Diazepam:* 58.83 ± 2.31) (Fig. 4b, *Diazepam*). Strikingly, similar to its protective effects on anxiety-like behaviour, oral gavage of vehicle *also* prevented the delayed BLA spinogenesis (*Control + Vehicle:* 72.41 ± 2.72; *Stress + Vehicle:* 63.81 ± 23.53) (Fig. 4b, *Vehicle*). However, the decrease in spine density due to diazepam was significantly greater as compared to the vehicle-treated control animals (Fig. 4b). This was reflected as a main effect of diazepam (F_(1,112)_ = 7.45, *p* < *0.01*) in a two-way repeated measures ANOVA. There was also a main effect of stress (F_(1,112)_ = 4.89, *p* < *0.05*), although there was no interaction between both factors (F_(1,112)_ = 0.84, p = 0.36).

We next compared spine density in stressed and control animals which received oral gavage with animals which did not receive any pharmacological manipulation (Fig. 2, referred to here as the ‘no gavage’ group). Analysis by a 2 × 3 ANOVA also showed a significant main effect of gavage (F_(2,160)_ = 59.27, *p* < *0.0001*), without any effects of stress (F_(1,160)_ = 0.35, p = 0.06). However, there was a significant interaction between both factors (F_(2,160)_ = 8.72, *p* < *0.001*), implying that oral gavage, irrespective of its nature, altered the trajectory of spine density changes elicited by stress. Indeed, BLA dendritic spine density in stressed animals which did not receive gavage was significantly higher than both the control (no gavage) group as well as all animals which received gavage, in support of this (Table 1, *BLA spine density*). Therefore, in addition to the protective effects of diazepam, post-stress vehicle administration alone, quite surprisingly, prevented the delayed effects on BLA spinogenesis.

### 3.5 Oral gavage of vehicle enhances corticosterone levels comparable to those caused by acute stress

Next we probed the counterintuitive protective effects of a single dose of vehicle against the delayed effects of acute immobilisation. One explanation for this could be that the process of vehicle gavage administration is stressful in itself, leading to an increase in corticosterone levels (Brown et al., 2000; Walker et al., 2012). This increased levels of corticosterone could, in turn, mitigate the initial effects of acute immobilisation stress that preceded oral gavage. In fact, elevating corticosterone levels *before* acute immobilisation stress has previously been shown to prevent the delayed increase in anxiety and BLA spinogenesis 10 days later (Rao et al., 2012). In the present study, could a similar surge of corticosterone, even *after* stress, also exert a protective effect? To address this possibility, we tested if oral gavage of vehicle elevates corticosterone levels.

**Fig. 5:**
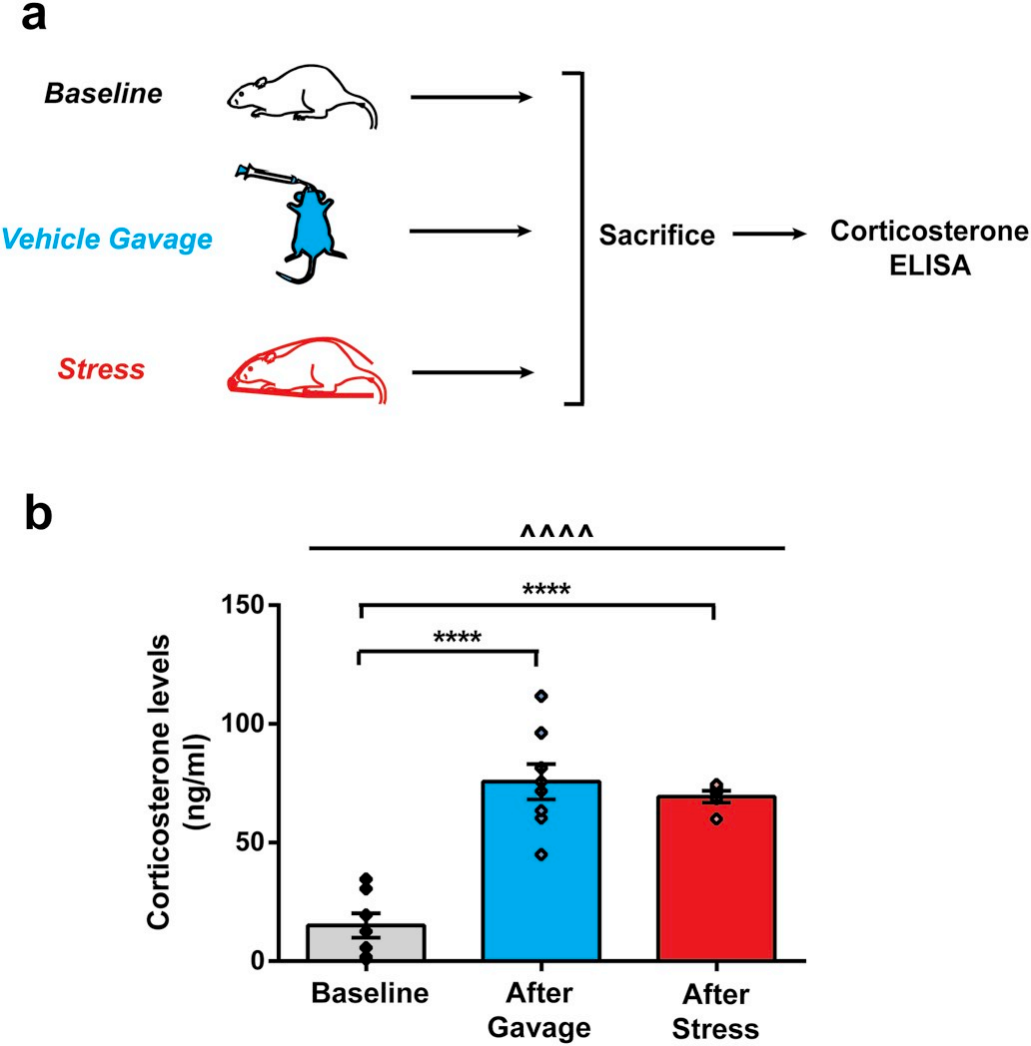
**Oral gavage of vehicle is stressful to rats. (a)** Experimental design. Different cohorts of rats were sacrificed after handling (baseline), after vehicle gavage and after acute immobilisation stress, and corticosterone levels were quantified subsequently. **(b)** Oral gavage of vehicle as well as stress significantly increases corticosterone levels. Increase in corticosterone due to gavage and stress were not significantly different from each other (Baseline, N = 7, After Gavage, N = 8, After Stress, N = 5). ^^^^ indicates p < 0.0001 in One-way ANOVA. **** indicate p < 0.0001 in *post-hoc* Tukey's test.

In a separate cohort of unstressed rats, oral gavage of vehicle caused a significant increase in serum corticosterone, compared to baseline unstressed levels (*Baseline:* 15.10 ± 5.14 ng/ml, *Vehicle Gavage:* 75.65 ± 7.43 ng/ml) (Fig. 5b, *after gavage*). Was this increase in corticosterone comparable to that triggered by the 2-h acute immobilisation stress alone? To answer this, a separate group of young rats were sacrificed after 2-h immobilisation stress, and serum corticosterone levels were assayed. Indeed, the rise in corticosterone triggered by acute stress was not different from levels elicited by oral gavage of vehicle (*Stress:* 69.34 ± 2.53 ng/ml*, Vehicle Gavage:* 75.65 ± 7.43 ng/ml) (Fig. 5b). Comparing all three groups by a one-way ANOVA also revealed statistically significant difference between them (F_(2,17)_ = 30.39, *p* < *0.0001*). Taken together, these results suggest that not only was oral gavage as stressful as immobilisation stress itself, but gavage-induced corticosterone 1 h after immobilisation stress was most likely also able to prevent the delayed increase in anxiety-like behaviour and BLA spines.

## 4 Discussion

The present study was aimed at preventing the delayed effects of acute immobilisation by intervening *after* stress exposure in young rats. We first confirmed earlier findings that 2 h of acute immobilisation stress increases anxiety-like behaviour on the elevated plus-maze and enhances BLA spinogenesis 10 days later. These delayed effects were prevented by a single oral dose of diazepam administered 1 h *after* immobilisation stress. Strikingly, we also observed a protective effect of vehicle gavage on these delayed effects, in the complete absence of diazepam. Oral gavage of vehicle was found to elicit an increase in corticosterone comparable to that due to stress. Together, these findings suggest that a surge in corticosterone elicited by the gavage procedure, after immobilisation stress, prevented its delayed detrimental effects.

Our results are in agreement with an earlier study wherein administration of the benzodiazepine alprazolam 1 h *after* acute predator scent stress prevented the increase in anxiety-like behaviour on the elevated plus-maze 4 days later (Matar et al., 2009). Our observations suggest that not only do such protective effects last up to 10 days after stress, it also prevents delayed BLA spinogenesis. Both these delayed effects of immobilisation stress were, surprisingly, also prevented by elevated corticosterone levels caused by vehicle-gavage 1 h after the stress. These findings add to previous evidence that oral gavage administration is indeed stressful, elevating both systemic levels of corticosterone (Brown et al., 2000), as well as excreted corticosterone metabolites (Walker et al., 2012). Notably, such counterintuitive protective effects of glucocorticoids have also been reported in clinical studies. For example, hydrocortisone administration in trauma victims, after the trauma, has been shown to prevent the subsequent development of PTSD symptoms (Schelling et al., 2006, 2001; Zohar et al., 2011). Further, detrimental effects of a recent traumatic event were occluded in patients who had a prior history of trauma exposure (Resnick et al., 1995), suggesting the protective effect of a previous stressful experience. These observations have been extended to the preclinical context as well. In fact, corticosterone administration for a duration of 12-h *before* acute immobilisation stress has been reported to prevent the delayed enhancement in anxiety-like behaviour and BLA spinogenesis (Rao et al., 2012). The present observations suggest the possibility that elevating corticosterone even *after* immobilisation has a similar protective effect, thereby extending this window-of-opportunity for interventions against stress.

Interestingly, an earlier study reported that hydrocortisone administration 1 h after predator scent stress prevented the delayed increase in anxiety-like behaviour on the elevated plus-maze and dendritic spine loss in the dorsal dentate gyrus (Zohar et al., 2011). Apart from the dentate gyrus, anxiety-like behaviour is also regulated by the BLA (Chattarji et al., 2015), such that behavioural and genetic manipulations affect both morphological plasticity in BLA and anxiety-like behaviour in the same direction. For example, overexpression of brain-derived neurotrophic factor in the BLA leads to enhanced spine density in BLA neurons as well as elevated anxiety-like behaviour, mimicking the effects of stress (Govindarajan et al., 2006). On the other hand, antidepressant treatment that prevents chronic stress induced hypertrophy of BLA neurons also prevents enhanced anxiety (Pillai et al., 2012). Together, these observations have identified stress-induced structural changes in the BLA as potential neural correlates of enhanced anxiety-like behaviour. In the present study, this was successfully reversed by both an anxiolytic drug as well as by gavage-induced increase in corticosterone levels. Interestingly, acute stress specifically increases the density of mature mushroom spines on BLA pyramidal neurons (Maroun et al., 2013). Whether intervention strategies undertaken in the present study also affect distributions of specific dendritic spine subtypes remains to be explored, and might provide greater insights into the micro-architectural changes within the BLA underlying stress-induced amygdalar dysfunction.

While both these above strategies reverse BLA spinogenesis, what could be the possible mechanisms by which each manipulation attains this? One possibility is that diazepam acts by potentiating inhibition at the GABA_A_ receptors (Xu and Sastry, 2005). Stress exposure is known to tilt the balance in favour of increased excitation in BLA, while simultaneously impairing inhibition (Duvarci and Pare, 2007; Suvrathan et al., 2014; Yasmin et al., 2016). For example, not only does chronic immobilisation stress strengthen long-term potentiation (LTP) in BLA principal neurons (Suvrathan et al., 2014), acute immobilisation stress also leads to an increase in BLA excitatory currents 10 days later (Yasmin et al., 2016). In addition, stress (Suvrathan et al., 2014) and corticosterone treatment (Duvarci and Pare, 2007) both reduce GABAergic inhibition in the BLA. Under such physiological conditions, diazepam might exert its anxiolytic effects by directly acting on GABA_A_ receptor subunits (Babaev et al., 2018), thereby counteracting the stress-induced increase in BLA excitation. Further, increasing inhibition could be a convergent strategy by which both diazepam, as well as corticosterone (elicited by vehicle gavage) might exert their protective effects. A seminal study by Karst et al. (2010) has shown that treating BLA principal neurons with corticosterone 30 min after acute restraint stress prevented stress-enhanced glutamate release via activation of the endocannabinoid pathway (Joëls et al., 2012; Karst et al., 2010). Future studies will be needed to examine if the two surges of corticosterone (caused by immobilisation, followed by oral gavage) might have mitigated the delayed effects of acute immobilisation stress through a similar mechanism. Interestingly, endocannabinoids, similar to diazepam, can also directly potentiate activity of GABA_A_ receptors (Sigel et al., 2011). Administration of endocannabinoid receptor agonists together with diazepam not only has a synergistic anxiolytic effect on the plus-maze (Naderi et al., 2008) but it also reduces serum corticosterone levels (Saber-Tehrani et al., 2010). Could this explain the significantly reduced spine density changes upon oral gavage of diazepam? One strategy to address this dichotomy is to administer benzodiazepine via drinking water. This would ensure that the protective effects of oral gavage does not occlude the anxiolytic effects of diazepam. Such studies would also enable us to further investigate if potential cellular mechanisms underlying the protective effects of both diazepam and corticosterone treatment converge, for example, on the endocannabinoid signalling pathway – a possibility that needs to be explored further.

## 5 Conclusion

In summary, our experiments show that oral gavage, both with and without an anxiolytic drug, can reverse anxiety-like behaviour as well as increased spine density in the BLA principal neurons. These observations also point to future directions of enquiry. For instance, does the post-stress intervention window extend *beyond* 1 h? If so, how late after stress will such intervention still be effective in rolling back the delayed impact of stress on the amygdala? How long lasting are these preventive effects? Also, do the potential benefits of such post-stress interventions extend to other forms of anxiety-like behaviour, such as social avoidance? Finally, what are the molecular and synaptic mechanisms by which these protective effects are achieved? Answers to these questions will offer valuable insights into the structural and functional effects triggered by traumatic stress over time, and their reversal using clinically effective therapeutic interventions.

## Conflicts of interest

The authors declare no conflict of interests.

## Author contributions

PC and SC contributed to the experimental design. PC performed the experiments and analysed the data. PC and SC interpreted the results. PC and SC wrote the manuscript.
